# Gastrointestinal Cancers: Molecular Genetics and Biomarkers

**DOI:** 10.1155/2018/4513860

**Published:** 2018-10-18

**Authors:** Kiran L. Sharma, Vikram Bhatia, Prasoon Agarwal, Ashok Kumar

**Affiliations:** ^1^Department of Urology, Mayo Clinic, 200 1st St. SW, Rochester, MN 55905, USA; ^2^Department of Oral Biology, College of Dentistry, University of Manitoba, 780 Bannatyne Avenue, Winnipeg, MB R3E0W2, Canada; ^3^Department of Pharmacology and Therapeutics, University of Manitoba, Winnipeg, MB R3E 3P4, Canada; ^4^Department of Surgical Gastroenterology, Sanjay Gandhi Post Graduate Institute of Medical Sciences, Lucknow 226014, India

Cancer is one of the main primary reasons of numerous disorders, disabilities, and mortality, around the globe. Gastrointestinal (GI) cancer is one of foremost health concerns in the world between all cancers and the loads are growing in several nations [1]. Most of the cancers are pathologically heterogeneous and triggered sequential build-up of variations in the genome that controls the normal physiology of the cell. It might be possible in the recent genomic era that the use of genomic biomarkers will be developed as significant genetic tools used by physicians for estimation of detection of cancer risk at microscopic levels and define the utmost suitable treatments for GI malignancies and also to screen the efficiency of suitable therapies [2]. Microsatellite Instability (MSI) caused by DNA mismatch repair deficiency is a significant prognostic as well as predictive biomarker in GI Cancers [3, 4]. Moreover, tumor genotyping [5] and signalling pathways like RAS/BRAF [6], PI3K [7], WNT, and ß-catenin stat3 have significant role in GI cancers [8, 9].

The genomic and epigenomic biomarker discoveries with the use of high throughput technologies can lead to discovery of molecular targets and early detection of cancer. It is established that like the genetic abnormalities, epigenetics events are also potentially responsible for cancer initiation and progression and DNA methylation is one such epigenetic modification. In DNA methylation, the cytosine preceding a guanine in the DNA is methylated. It exists in two states: hyper methylation (methylation is increased) and hypomethylation (methylation is reduced). Different cancer types exhibit exclusive pattern of methylation changes in their genome. DNA methylation has huge potential to be used as biomarkers which can be used to improve the diagnosis, screening, prognosis, and prediction for treatment. Genome wide studies (GWAS) on DNA methylation have helped in revealing the novel biomarkers as well as different subgroups. This special issue summarizes the current understanding of the molecular pathogenesis of the GI cancers and focused on exploring the genomic, biochemical, and epigenomic impact on gastrointestinal oncology.

E. Fredericks et al. study in the present issue has examined and compared *β*-catenin, PPAR*γ*, COX- 2, and IL-17A in the colonic mucosa of CRC, irritable bowel syndrome (IBS), and inflammatory bowel disease (IBD) patients, alongside a normal population and identified important role of *β*-catenin as major driver of colorectal carcinogenesis but is controlled by many more players other than APC. This study also found PPAR*γ* elevation showing an anticarcinogenic effect. The authors suggest that* COX-2 *expression at posttranscriptional regulation needs to be further evaluated in colorectal cancer.

J. Yao et al. aimed to explore the Circulating Tumor DNA Mutations (ctDNA) by genotyping ctDNA by next-generation sequencing (NGS) based panel of 40 genes to categorize the prognostic mutation status in the tumor tissue of 76 metastatic colorectal cancer (mCRC) patients and chemotherapeutic response. The study suggested the prognostic role of mutations in* RAS/BRAF *genes identified in ctDNA predict as worse progression-free survival (PFS) among these patients.

N. Currey et al. in their present paper elaborated the importance of MSI in animal models of colorectal cancer (CRC). The authors compared knockout of Msh2 and p53 gene and wild type mice treated with/without carcinogen azoxymethane (AOM) and sulindac (anti-inflammatory nonsteroidal drug) followed by analysing of a panel of 5 mononucleotide repeat markers in normal colon tissue and induced tumors. They found MSI identified all the way through in control colon in untreated Msh2-KO mice involving contraction of the repeat sequences compared to WT. The longer mononucleotide repeats markers found were the most profound for MSI whereas markers with shorter repeats presented slight change only. The AOM exposure further caused reduction of Bat37 and Bat59 repeats in the distal part of colon of Msh2-KO mice. AOM exposure additionally caused narrowing of Bat37 and Bat59 repeats and this effect was shown to be reversed by sulindac. The authors further concluded tumorigenesis induced by AOM is linked to greater MSI in Msh2-KO mice colon but not in WT/p53-KO mice and sulindac treatment prevented associated increased MSI.

I. S. Yu and W. Y. Cheung have provided a comprehensive review about role of predictive/prognostic biomarkers and treatment background in adenocarcinoma of pancreas disease. The authors emphasized that existing biomarkers have absence of optimization across medical practices and concluded an overview of the current treatment possibilities regarding pancreatic cancer and enlightened about newer visions into the present landscape, future guidelines, and directions for biomarker discovery for pancreatic adenocarcinoma progression and disease development.

J. Maues et al. studied epigenetic regulation of MYC-regulated expression patterns in three Brazilian gastric cancer cell lines representing different histological subtypes (diffuse, intestinal, and metastasis) of gastric cancer (GC). By using siRNA and Next Generation Sequencing (NGS) approaches, authors identified MYC-regulated differentially expressed genes (DEGs) profile in gastric cancer cell lines which share a common core of deregulated genes. This study can help in step further understanding of MYC's mediated regulation role in GC and might help in developing new therapeutic targets in GI cancers.

Articles published in this present special issue have highlighted the importance of the molecular mechanism associated with GI cancers by their own individual studies. GI cancers are one of the polygenic multifactorial and complex diseases with epigenetics, genetic, and environmental factors all playing important role. Still a lot need to be researched to understand the molecular mechanism associated with GI cancer so that markers for early detection could be developed and the disease can be treated at an early stage which is an important requisite for better outcome.
